# Disc-Hub: a python package for benchmarking machine learning strategies in DIA-MS identification

**DOI:** 10.1093/bioadv/vbaf232

**Published:** 2025-09-30

**Authors:** Yiwen Yu, Xiaohui Wu, Jian Song

**Affiliations:** Cancer Institute, Suzhou Medical College, Soochow University, Jiangsu 215123, China; Cancer Institute, Suzhou Medical College, Soochow University, Jiangsu 215123, China; Cancer Institute, Suzhou Medical College, Soochow University, Jiangsu 215123, China

## Abstract

**Motivation:**

Accurate analysis of data-independent acquisition (DIA) mass spectrometry data relies on machine learning to distinguish target peptides from decoy peptides. Different DIA identification engines adopt distinct binary classifiers and training workflows to accomplish this learning task. However, systematic comparisons of how different machine learning strategies affect identification performance are lacking. This absence of evaluation hinders optimal learning strategy selection, increases the risk of model underfitting or overfitting, and ultimately undermines the effectiveness and reliability of false discovery rate (FDR) control.

**Results:**

In this study, we benchmarked three training strategies and four classifiers on representative DIA datasets. Among them, K-fold training combined with a multilayer perceptron achieved the best balance between identification depth and FDR control. We have released the datasets and code through the Python package Disc-Hub, enabling rapid selection of optimal machine learning configurations for developing DIA identification algorithms.

**Availability and implementation:**

Disc-Hub is released as an open source software and can be installed from PyPi as a python module. The source code is available on GitHub at https://github.com/yuyiwen-yiyuwen/Disc_Hub.

## 1 Introduction

Data-independent acquisition (DIA) mass spectrometry, fragmenting all precursors within predefined *m*/*z* windows, has emerged as a crucial technique in proteomic research (Gillet *et al.* 2012). Compared to data-dependent acquisition (DDA), DIA demonstrates superior performance in terms of peptide detection reproducibility and depth, which has facilitated the widespread adoption of this technique in biomarker discovery, clinical cohort studies, and single cell proteomic analyses (Guo *et al.* 2025). However, the characteristics of the DIA method cause the coupling of fragment ion signals, and its MS/MS spectra originate from multiple peptide ions, which poses a significant challenge to peptide identification algorithms based on DIA data.

A typical peptide-centric DIA identification workflow generally consists of three stages: (i) constructing a spectral library, which provides target and decoy peptide sets, along with peptide chromatographic and mass spectrometric properties (e.g. retention time, fragment ion intensities); (ii) extracting the peptide chromatograms based on the information from the spectral library and scoring the potential peak groups across multiple dimensions (e.g. retention time deviation, mass deviation); (iii) discriminating target peptides from decoy peptides using a machine learning classifier (i.e. a discriminator) based on these scores, followed by statistical validation based on the decoy peptides, and reporting of statistical analysis results (Röst *et al.* 2014). It is evident that the specific implementation of the discriminator clearly impacts the identification results in DIA. mProphet was the first algorithm to introduce a semi-supervised learning strategy for the analysis of targeted mass spectrometry data (Reiter *et al.* 2011). It treats all decoy peptides as negative samples for training the discriminator, while target peptides are regarded as unlabeled or unknown samples. Through iterative training of a linear discriminant analysis (LDA) model, mProphet progressively expands the set of positive samples from the pool of unknowns in each iteration, thereby continuously improving discriminating performance. OpenSWATH (Röst *et al.* 2014), as the first comprehensive DIA engine, adopted the semi-supervised learning approach from mProphet and re-developed PyProphet (Teleman *et al.* 2015). EncyclopeDIA (Searle *et al.* 2018) retains the semi-supervised learning training strategy, but the discriminator model uses an support vector machine (SVM). DIA-NN (Demichev *et al.* 2020) and Beta-DIA (Song *et al.* 2024), achieve substantial improvements in DIA identification by using fully supervised learning—treating all target peptides as positive samples—in combination with ensemble neural network-based discriminators. Since fully supervised learning involves a complete overlap between the training and test sets, it poses a higher risk of overfitting compared to semi-supervised approaches. To mitigate this risk, both tools limit the training of the discriminator to a single iteration. In contrast, MaxDIA (Sinitcyn *et al.* 2021) addresses overfitting by using a training scheme similar to K-fold cross-validation, in which models are trained on in-fold data and generate predictions on out-of-fold data (throughout the text, “K-fold” refers to this training approach). This 5-fold training is further coupled with the XGBoost classifier, which is considered to offer superior discriminative power compared to neural networks. Similarly, Dream-DIA (Gao *et al.* 2021) trains its discriminator using a fully supervised learning framework combined with XGBoost.

Obviously, the performance of a discriminator depends not only on its training strategy—such as semi-supervised learning, fully supervised learning, or K-fold training—but also on the choice of the specific binary classifier. Although the above-mentioned tools have conducted preliminary comparisons of how different models affect identification results in their respective publications, these comparisons still lack comprehensiveness, systematicness and thoroughness. Comprehensiveness refers to the need to explore as many combinations of training strategies and classifier choices as possible. Systematicness implies that evaluations should cover a wide range of test datasets, particularly those with low detections—such as plasma, high-throughput, and single-cell data—which are more prone to overfitting for machine learning. Thoroughness means the need for in-depth analysis of the core issue of underfitting and overfitting, so as to avoid overly conservative identification caused by underfitting, as well as overly aggressive results due to overfitting (which makes the false discovery rate control invalid). The lack of evaluations that are comprehensive, systematic, and thorough has not only led to inconsistent conclusions regarding the application of machine learning across different tools, but may also prevent identification software from achieving optimal performance.

In light of this, we propose representative DIA datasets and evaluate the performance of various discriminator models under different training frameworks. To assess the degree of underfitting and overfitting—reflected by the number of identifications and the false positive rate—we use widely used and conceptually intuitive entrapment-based statistical evaluation methods to estimate a lower bound for the false discovery rate (FDR), defined as the ratio of the number of entrapment hits to the number of all hits (Wen *et al.* 2025). Through this comprehensive, systematic, and in-depth comparative analysis, we aim to provide practical insights and guidance for the application of machine learning in the development of DIA identification tools. All related code is publicly available and can be conveniently installed to enable reproduction of our results, evaluation on custom scoring strategies, and extension to other training methods and models.

## 2 Results

### 2.1 Datasets

To ensure the systematic evaluation of machine learning approaches, this study selected four representative diaPASEF (Meier *et al.* 2020) datasets—HeLa-QC, high-throughput, plasma, and single-cell samples (Table 1, available as supplementary data at *Bioinformatics Advances* online). The choice of diaPASEF data was motivated by its additional ion mobility dimension compared to conventional DIA data, resulting in higher-dimensional input features that provide a more rigorous test of machine learning capabilities. All four datasets were analyzed using Beta-DIA (Song *et al.* 2024) to generate discriminative sub-scores. Beta-DIA was chosen because it integrates both traditional functional scoring and deep learning-based scoring, offering one of the most comprehensive scoring designs currently available.

Specifically, this study initially used DIA-NN (version 2.0) to generate predicted spectral libraries from Homo sapiens (20 420 sequences, 06.2024) and Arabidopsis thaliana (16 310 sequences, 06.2024) fasta files. Predicted libraries were chosen over experimental ones because of their broader coverage in theory and the convenience of not requiring additional experimental acquisition, which enables a more comprehensive evaluation of training strategies and model performance. The digest params were: peptide length 7–30, charge states 1–4, maximum number of variable modifications 1 (oxidation on methionine). As a result, the final spectral library contained 9 557 086 precursors. In Beta-DIA, both human and Arabidopsis peptides are treated as target peptides, and each target peptide is paired with one decoy peptide using the mutate method, which substitutes terminal-adjacent amino acids based on the GAVLIFMPWSCTYHKRQEND → LLLVVLLLLTSSSSLLNDQE pattern. This decoy generation approach also ensures a 1:1 ratio of targets to decoys. After generating 793 sub-scores for each target and decoy peptide, Beta-DIA applies machine learning to distinguish between them and calculates the decoy-based FDR. Storing all precursors and their scores is neither necessary—many exhibit zero or low-quality signals—nor practical, as the full dataset (∼10 million targets and ∼10 million decoys with associated scores) could exceed 60 GB (assuming each score occupies 4 bytes). Therefore, following DIA-NN’s initial filtering threshold, Beta-DIA retained all human, Arabidopsis, and decoy peptides with FDR < 0.5, along with their sub-scores, as the test dataset, saved in TSV format. The inclusion of Arabidopsis peptides among the target peptides allows for the estimation of the actual FDR. The discrepancy between the actual FDR and the reported decoy-based FDR serves as an indicator of underfitting or overfitting of the discriminator model.

### 2.2 Metrics

This paper refers to the FDR defined by Arabidopsis as the external FDR or real FDR and the FDR defined by decoy as the reported FDR. The performance disparity among different models is primarily reflected in two aspects: the consistency between external FDR (1%–5%) and reported FDR (1%–5%), serving as a measure of the degree of model underfitting and overfitting; The second point concerns the number of identifications under the external 1% FDR. Only when the external FDR closely matches the reported FDR can a higher identification at external FDR level be regarded as truly beneficial.

### 2.3 Methods

**Figure 1.**
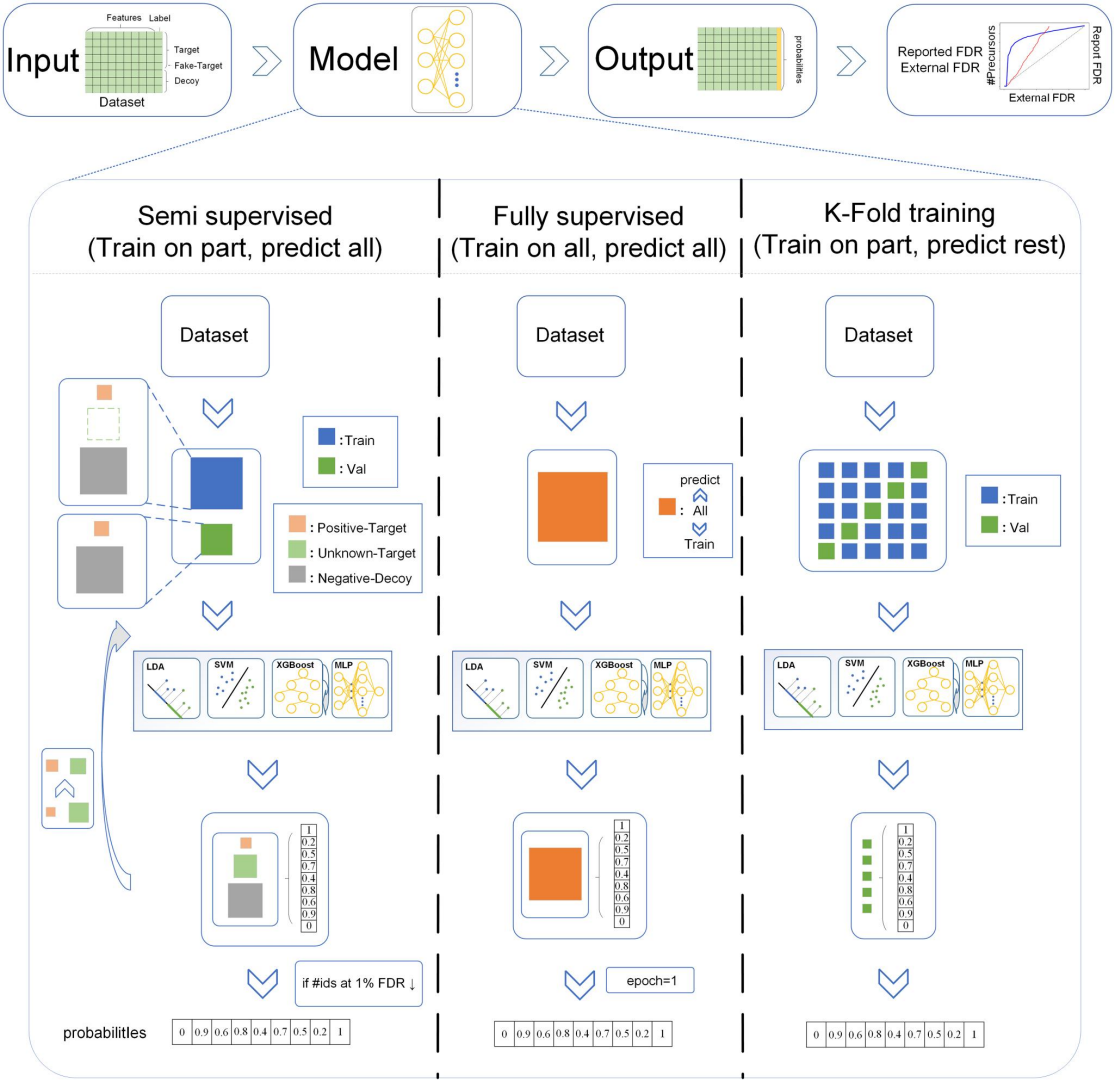
The workflow of Disc-Hub. “Train on part, predict all” refers to using high-confidence target samples as positive samples, with the trained model making predictions on all target samples (including those used for training) and all decoy samples. “Train on all, predict all” refers to using all target samples as positive samples, with the trained model making predictions on all target samples (including those used for training) and all decoy samples. “Train on part, predict rest” refers to using a subset of target samples as positive samples, with the trained model making predictions on the remaining target samples and all decoy samples. In all cases, decoy samples are treated as negative samples.

The implementation of a discriminator involves two key components: the training framework and the choice of classification model (Fig. 1). This study compares three training frameworks: semi-supervised learning, fully supervised learning, and K-fold training. All three frameworks treat decoy peptides as negative samples during training, with the primary distinction being how many target peptides are used as positive samples. The semi-supervised framework uses the fewest positive samples (although the positive sample set expands during the iterative training) and allows multiple-epoch training. The fully supervised framework uses all target peptides as positive samples but limits the number of training epochs to one in order to avoid potential overfitting. The K-fold (default 5-fold) training strategy uses 80% of the target peptides as positive training samples while completely isolating the test set from the training set, thereby balancing the trade-off between the quantity of positive samples and the number of training iterations. Details of the implementation for each training framework are provided in Note 1, available as supplementary data at *Bioinformatics Advances* online.

For classification model selection, we considered four types of classifiers: LDA, SVM, XGBoost, and ensemble multilayer perceptrons (MLPs). Model-specific parameters were empirically tuned to maximize learning performance, except for the MLPs model, which directly adopted the same parameter settings as those used in DIA-NN. Detailed configurations of each model are provided in Note 2, available as supplementary data at *Bioinformatics Advances* online.

### 2.4 Performance

We evaluated the performance of different combinations of training frameworks and models on the test datasets. Under the semi-supervised framework (Fig. 1, available as supplementary data at *Bioinformatics Advances* online), both XGBoost and MLPs exhibited substantial overfitting in single-cell and plasma datasets, as evidenced by actual FDRs exceeding the reported FDR. While LDA and SVM did not show signs of overfitting under the same framework, their identification yields were significantly lower than those of XGBoost and MLPs. When using the fully supervised framework (Fig. 2, available as supplementary data at *Bioinformatics Advances* online), LDA, XGBoost, and MLPs (fully supervised training with MLPs, mirroring the configuration of DIA-NN) again showed overfitting on the plasma dataset, with actual FDRs exceeding the nominal 1% threshold. Although SVM maintained proper FDR control under this condition, it produced the lowest number of identifications at the same actual FDR. In contrast, the K-fold training framework (Fig. 3, available as supplementary data at *Bioinformatics Advances* online) consistently prevented overfitting across all models and datasets, with actual FDRs remaining below the reported values. Moreover, the number of identifications achieved with K-fold training was comparable to those obtained under the semi- and fully supervised frameworks.

These results suggest that the semi-supervised and fully supervised frameworks, which do not enforce strict separation between training and test sets, are prone to data leakage. In light of this, more expressive models such as XGBoost and MLPs are more susceptible to overfitting, whereas simpler models like LDA and SVM are less prone to overfitting but may underfit the data, resulting in fewer identifications. By contrast, K-fold training ensures rigorous separation between training and test data, effectively preventing overfitting regardless of the learning algorithm used, while achieving identification performance comparable to the other frameworks. Specifically, under the K-fold framework, all four models achieved similarly well-controlled FDRs. The ranking of identification yield at both the reported 1% FDR and actual 1% FDR was: MLPs > XGBoost > SVM and LDA.

In summary, the combination of K-fold training and MLPs offers both strict FDR control and depth of coverage. This balance makes it particularly suitable for applications in proteomics that demand both high accuracy and sensitivity.

## 3 Discussion

The machine learning task for DIA identification exhibits certain unique characteristics that distinguish it from conventional machine learning problems. In standard settings, the training and testing datasets are strictly separated to avoid information leakage. However, in DIA identification, both training and testing are performed on the same dataset. This presents a trade-off: overfitting leads to invalid FDR estimation, while underfitting results in insufficient peptide identifications.

Semi-supervised training expands the training set iteratively but begins with a minimal portion of the data, potentially causing inadequate global fitting. Fully supervised training leverages the entire dataset, but risks overfitting due to data leakage. To mitigate this, the training is limited to a single epoch, which may lead to underfitting. In contrast, the K-fold training strategy strictly separates training and testing sets to prevent leakage, and allows for multiple training epochs within each fold, addressing both overfitting and underfitting concerns. This makes K-fold training a potentially more suitable framework for DIA identification.

Regarding classifier choice, LDA and SVM lack the capacity to model non-linear feature relationships, which can limit identification depth. On the other hand, XGBoost and MLPs offer stronger nonlinear modeling capabilities, better representation of complex feature interactions, robustness through ensembling, and regularization mechanisms that make them well-suited for high-dimensional learning tasks. Nevertheless, XGBoost is sensitive to hyperparameters, and its performance can vary significantly across datasets unless carefully tuned—a process that is often challenging. MLPs, in comparison, demonstrate greater robustness. Therefore, a promising future direction for machine learning in this context is the development of automated model selection or hyperparameter optimization strategies, moving beyond the one-param-fits-all paradigm.

Another factor influencing model performance is dataset size. While training is conducted in mini-batches regardless of the training framework, the overall dataset size may have a limited effect on training frame. However, from the perspective of the classifier, larger datasets may help offset differences in model expressiveness. For example, on smaller-scale datasets such as single-cell and plasma samples, XGBoost and MLPs significantly outperform LDA and SVM in terms of identification depth. In contrast, on larger-scale datasets such as high-throughput and QC samples, the performance gap between models narrows. This suggests that data augmentation may be another valuable direction for improving machine learning strategies.

Theoretically, Disc-Hub is applicable to any search engines that uses a target–decoy strategy with multiple scoring features for peptide identification, including but not limited to DDA, DIA, plexDIA (Derks *et al.* 2023), and scanning SWATH (Messner *et al.* 2021) data. However, its current applicability is restricted to identification tools that generate intermediate results containing decoys and their associated scores. For tools without such support, further adaptations would be required. In practice, developers of search engines can enable Disc-Hub by exporting intermediate results with decoys and their associated scores, which would facilitate rapid benchmarking of training strategies and thereby accelerate tool development. We plan to extend Disc-Hub evaluations to a broader range of data types and identification tools in future work.

## Supplementary Material

vbaf232_Supplementary_Data

## Data Availability

The data underlying this article are available in https://figshare.com/articles/dataset/Disc_Hub/29163410.
