# Supplementary material for: Disc-Hub: a python package for benchmarking machine learning strategies in DIA-MS identification
Source: Bioinform Adv. 2025 Sep 30;5(1):vbaf232. doi: 10.1093/bioadv/vbaf232 (PMC12597894; doi:10.1093/bioadv/vbaf232)
Supplement: vbaf232_Supplementary_Data [file vbaf232_supplementary_data.docx]

Supplementary information

Table 1. Four representative MS datasets for benchmark.

| ID | Accession ID | File Name | Note |
| --- | --- | --- | --- |
| HeLa-QC | PXD017703 | 20211103_PRO2_LS_01_MA_HeLa_200_SDC_NS_RE2_1_1418 | Loading amount: 200 ng  Gradient length: 90 min |
| High Throughput | PXD017703 | 20200505_Evosep_200SPD_SG06-16_MLHeLa_200ng_py8_S3-A1_1_2737 | 200 samples per day (SPD) |
| Plasma | PXD047854 | PIQ001_EVOSEP01_TIMS04_PRO_HT_BOB2_NP5_Pooled_Loading_Curve_30SPD_0013_S3-C7_1_464 | Loading amount: 600 ng |
| Single cell | MSV000093867 | E_20221108_EvoSepOne_3rdGenAurora15cm_CC_40SPD_whisper_scLF1108_M10_S1-B2_1_3116 | A HEK-293T cell |

Supplementary Note 1

| **Semi Supervised** |  |
| --- | --- |
| **Input:**  dataset = features + label (target = 0, decoy = 1)  **Output:**  Final predicted probabilities on all samples  **Other called functions:**  cal_fdr(predicted_probabilities, label/ specie) → Reported FDR/External FDR  **Process:**  **1. Prepare data:**  X_total = features, y_total = 1 - label  Based on y_total:  Assign label = unknown to samples originally marked as target  Assign label = negative to samples originally marked as decoy  **2. Feature selection:**  For each feature_col in range(feature_cols):  feature_scores = X_total[:, feature_col]  Reported FDR = cal_fdr(feature_scores, label)  Choose the column with the best Reported 1% FDR  **3. Pseudo-label initialization:**  initial_target_indices = samples passing Reported 1% FDR and label == target  → Assign label = positive to initial_target_indices  permanent_negative_indices = samples where original label == decoy  → Assign label = negative to permanent_negative_indices  For remaining target samples (not selected):  → Assign label = unknown  **4. Split training data:**  X_train, y_train = 80% of initial_target + 80% of permanent_negative  X_val, y_val = 20% of initial_target + 20% of permanent_negative  **5. Iterative training:**  best_Reported_fdr = 0  **Loop:**  Train model using (X_train, y_train) and (X_val, y_val)  predicted_prob = model(X_total)  Reported 1% FDR = cal_fdr(predicted_prob, y_total)  If Reported 1% FDR < best_Reported_fdr:  Break  x_remaining = X_total - X_val, y_remaining = y_total - y_val  predicted_prob_remaining = model(x_remaining)  selected_target_indices = samples in x_remaining where:  predicted_prob passes Reported 1% FDR threshold and label == unknown  Assign new_label = positive to selected_target_indices  Add 80% of selected_target_indices to X_train / y_train  Add 20% of selected_target_indices to X_val / y_val | |

| **Fully Supervised** |
| --- |
| **Input:**  dataset = features + label (target = 0, decoy = 1)  epoch = 1  **Output:**  Predicted probabilities on all samples  **Other called functions:**  cal_fdr(predicted_probabilities, label/specie) → Reported FDR / External FDR  **Process:**  **1. Prepare data:**  X_total = features, y_total = 1 - label  **2. Train:**  Train model using (X_total, y_total) for 1 epoch  **3. Predict:**  predicted_probabilities = model(X_total) |

| **K-Fold Training** |
| --- |
| **Input:**  dataset = features + label (target = 0, decoy = 1)  K = 5  **Output:**  Predicted probabilities on all samples  **Other called functions:**  cal_fdr(predicted_probabilities, label/specie) → Reported FDR / External FDR  **Process:**  **1. Prepare data:**  X_total = features , y_total = 1 - label  **2. K-fold training:**  Initialize predicted_probabilities = 0  For fold in range(K):  Split X_total, y_total into:  X_train, X_val = 4:1 split for current fold  y_train, y_val = 4:1 split for current fold  Train model using (X_train, y_train) and (X_val, y_val)  predicted_probabilities += model(x_ val) |

Supplementary Note 2

**LDA**: In this work, the LDA model is completed using ‘*LinearDiscriminantAnalysis*’ class from scikit-learn (version 1.5.2), with all parameters set to their default values.

**SVM**: This discriminator employs a linear SVM with a maximum of 1,000 training iterations and a convergence threshold set at 0.001. To accommodate large-scale datasets and accelerate optimization, stochastic gradient descent (SGD) is utilized for approximate solutions. Probabilistic outputs are obtained by mapping decision scores to probabilities via the sigmoid function, and a 5-fold cross-validation is implemented during the calibration process to mitigate overfitting.

**XGBoost**: To balance comprehensive feature learning with overfitting prevention, the maximum depth of each tree was set to 6, and the learning rate was fixed at 0.1. The maximum number of training rounds was set to 300, with early stopping triggered if performance did not improve for 10 consecutive rounds. To accommodate large-scale datasets, the histogram-based algorithm was employed for model training.

**MLPs**: The MLPs model used in this study adopts the architecture of DIA-NN’s MLPs and is implemented as an ensemble of twelve MLP classifiers. Each classifier shares the same architecture, consisting of five hidden layers with 25, 20, 15, 10, and 5 neurons, respectively, and employs the ReLU activation function. To encourage model diversity, each MLP is initialized with an independent random seed. The models are trained using the Adam optimizer with a learning rate of 0.003 and a batch size of 50. To further regulate the training process, early stopping is optionally applied based on performance on a held-out validation subset comprising 10% of the training data. Training is terminated when no significant improvement is observed for five consecutive iterations, where improvement is defined as a minimum change of 1×10⁻⁴ in the monitored metric. In this study, early stopping is enabled under the semi-supervised and K-fold training frameworks, but disabled in the fully supervised setting. Additionally, the maximum number of training iterations is set to 100 for the semi-supervised and K-fold settings, and limited to a single iteration in the fully supervised setting to prevent overfitting. The final prediction is obtained by averaging the outputs of all individual models.

**
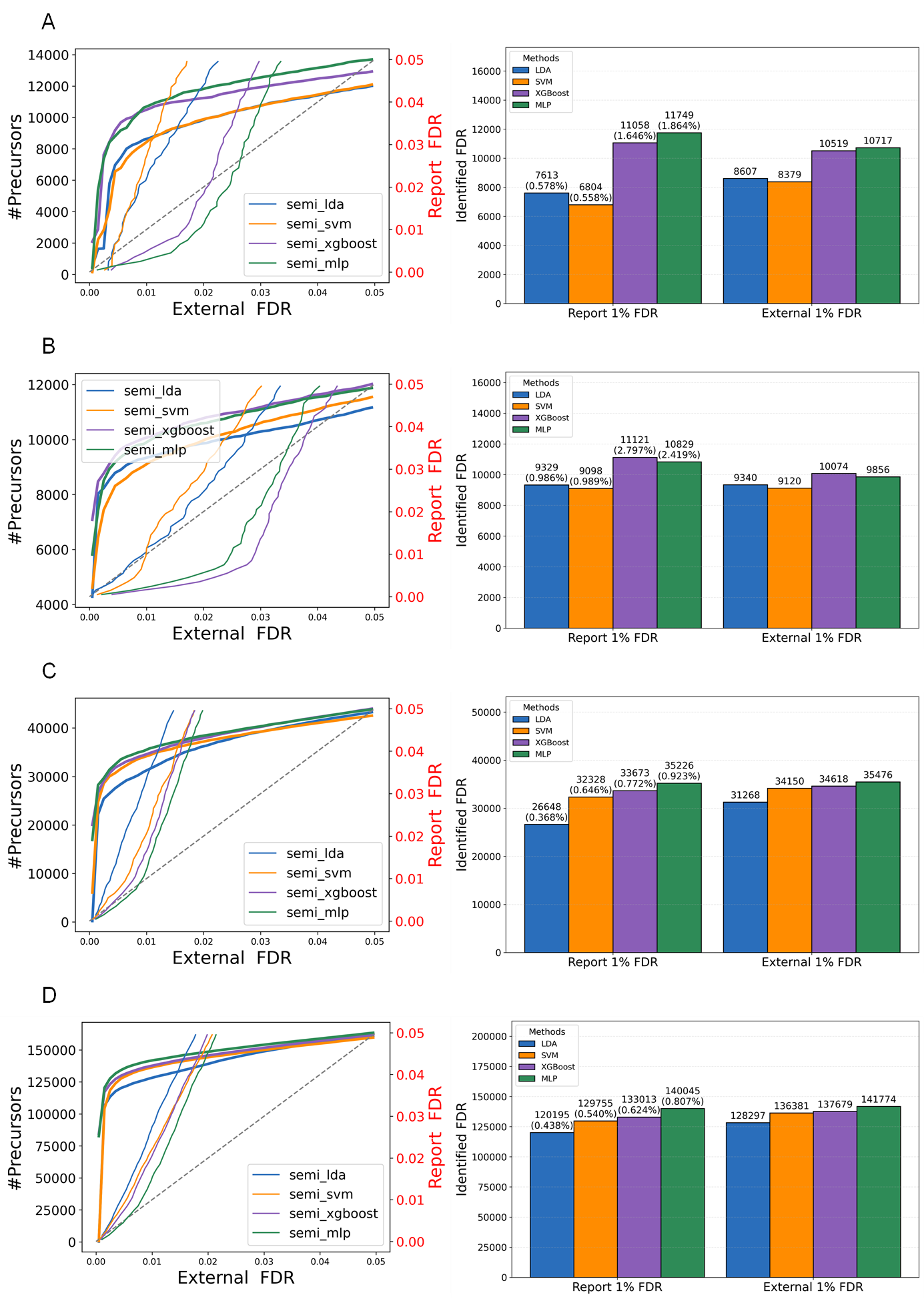
Figure 1.** Performance comparison of the four classification models with semi-supervised training workflow across all four datasets. **A)** On the single cell dataset. The left panel presents a dual Y-axis plot, with the external FDR on the X-axis, the number of identifications on the left Y-axis, and the Reported FDR on the right Y-axis. The right panel displays a bar chart, where the X-axis represents different discriminators and the Y-axis indicates the number of identifications. Dark bars denote external 1% FDR, while light bars represent Reported 1% FDR. The percentage values above the bars at Reported 1% FDR indicate the actual FDR under the Reported 1% FDR threshold, calculated as the number of *Arabidopsis* identifications divided by the total number of identifications at Reported 1% FDR. **B)** On the Plasma dataset. **C)** On the high-throughput dataset. **D)** On the Hela-QC dataset.

**
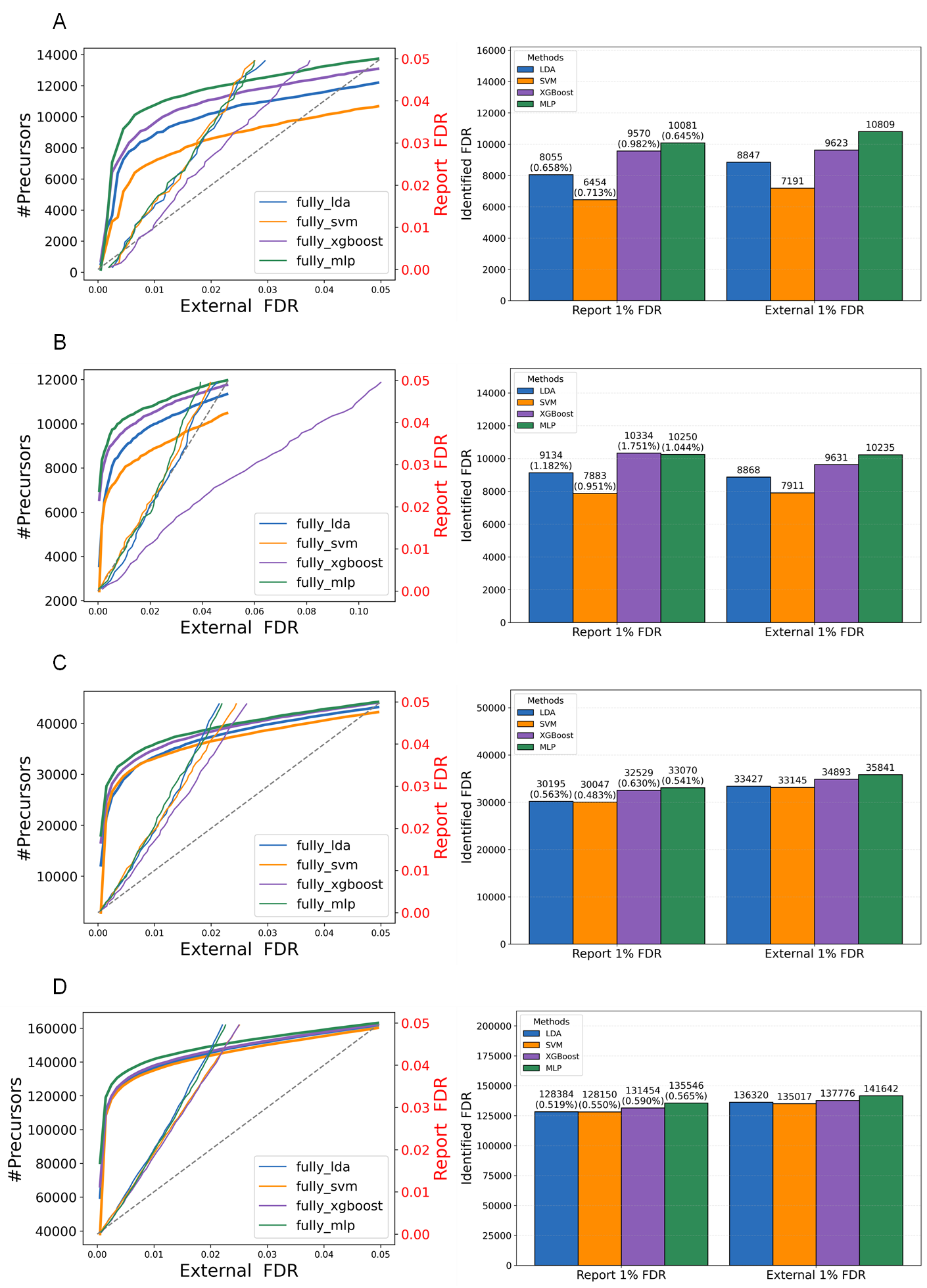
Figure 2.** Performance comparison of the four classification models with fully supervised training workflow across all four datasets. **A)** On the single cell dataset. The left panel presents a dual Y-axis plot, with the external FDR on the X-axis, the number of identifications on the left Y-axis, and the Reported FDR on the right Y-axis. The right panel displays a bar chart, where the X-axis represents different discriminators and the Y-axis indicates the number of identifications. Dark bars denote external 1% FDR, while light bars represent Reported 1% FDR. The percentage values above the bars at Reported 1% FDR indicate the actual FDR under the Reported 1% FDR threshold, calculated as the number of *Arabidopsis* identifications divided by the total number of identifications at Reported 1% FDR. **B)** On the Plasma dataset. **C)** On the high-throughput dataset. **D)** On the Hela-QC dataset.

**
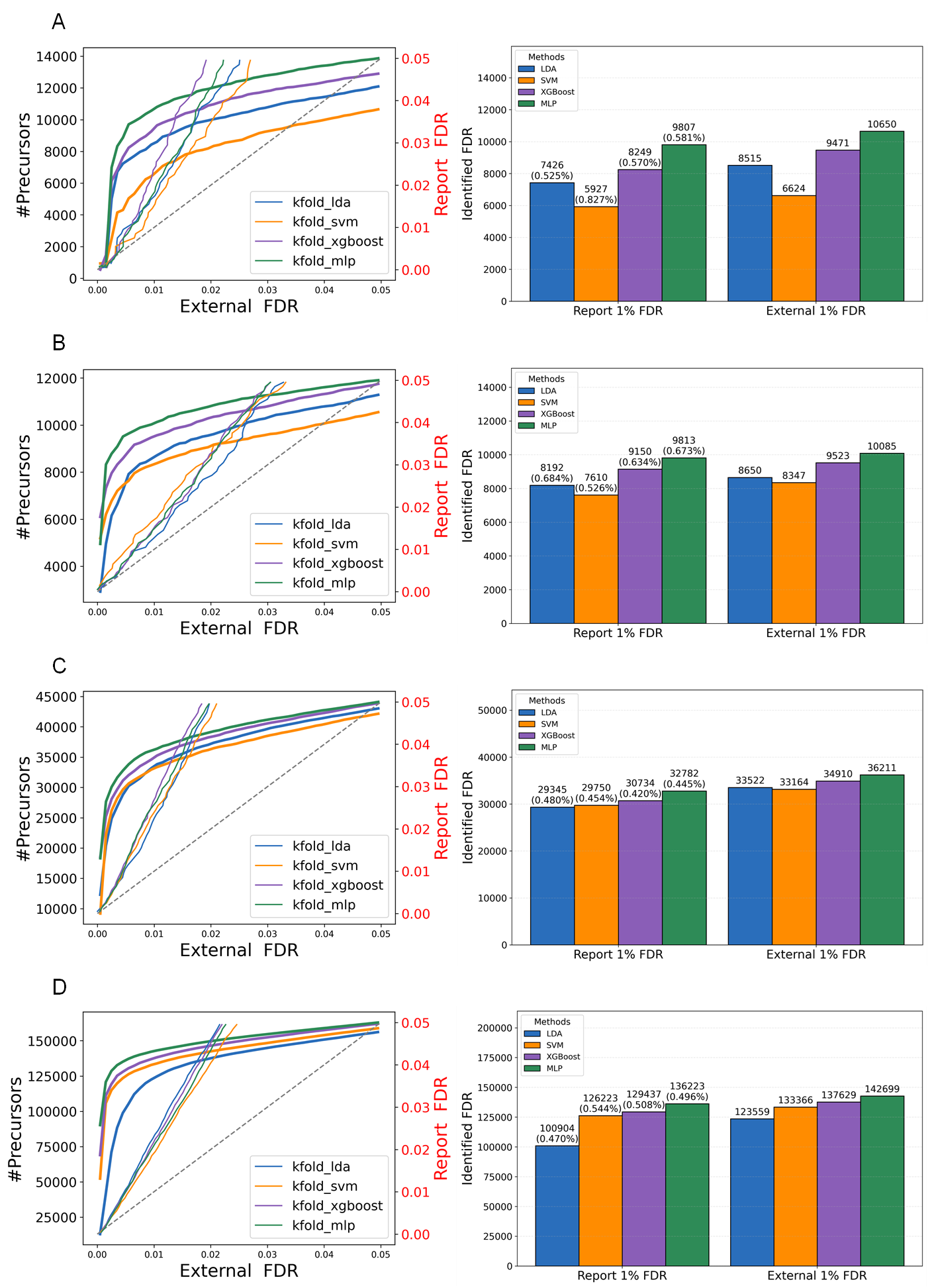
Figure 3.** Performance comparison of the four classification models with K-fold training workflow across all four datasets. **A)** On the single cell dataset. The left panel presents a dual Y-axis plot, with the external FDR on the X-axis, the number of identifications on the left Y-axis, and the Reported FDR on the right Y-axis. The right panel displays a bar chart, where the X-axis represents different discriminators and the Y-axis indicates the number of identifications. Dark bars denote external 1% FDR, while light bars represent Reported 1% FDR. The percentage values above the bars at Reported 1% FDR indicate the actual FDR under the Reported 1% FDR threshold, calculated as the number of *Arabidopsis* identifications divided by the total number of identifications at Reported 1% FDR. **B)** On the Plasma dataset. **C)** On the high-throughput dataset. **D)** On the Hela-QC dataset.
